# Milk Fat Globule-Epidermal Growth Factor-Factor 8 Reverses Lipopolysaccharide-Induced Microglial Oxidative Stress

**DOI:** 10.1155/2019/2601394

**Published:** 2019-03-13

**Authors:** Jie Li, Xiaotian Xu, Xiaoying Cai, Yinlun Weng, Yiping Wang, Qingyu Shen, Xiaolei Shi

**Affiliations:** ^1^Department of Neurology, Zengcheng District People's Hospital of Guangzhou, Guangzhou, China; ^2^Guangdong Key Laboratory for Diagnosis and Treatment of Major Neurological Diseases, Department of Neurology, National Key Clinical Department and Key Discipline of Neurology, The First Affiliated Hospital, Sun Yat-sen University, Guangzhou, China; ^3^Department of Anesthesiology, The First Affiliated Hospital, Sun Yat-sen University, Guangzhou, China; ^4^Department of Neurosurgery, Sun Yat-sen Memorial Hospital, Guangzhou, China; ^5^Department of Neurosurgery, The Fifth Affiliated Hospital, Sun Yat-sen University, Zhuhai, China; ^6^Department of Neurology, Yijishan Hospital, The First Affiliated Hospital of Wannan Medical College, Wuhu, China

## Abstract

Oxidative stress plays an important role in various neurological disorders. Milk fat globule-epidermal growth factor-factor 8 (MFG-E8) is a regulatory protein for microglia. However, its involvement in microglial oxidative stress has not been established. In this study, we observed microglial oxidative stress in response to lipopolysaccharide (LPS) both *in vitro* and *in vivo*. LPS induced significant elevation of TNF-*α*, IL-6, MDA, and ROS and reduction of GSH and SOD in the mouse brains and primary microglia, which were reversed by MFG-E8 pretreatment. MFG-E8 induced the expression of Nrf-2 and HO-1 that was reduced by LPS incubation. Moreover, LPS-increased Keap-1 expression was reversed by MFG-E8. But the above tendencies were not seen when MFG-E8 was applied alone. The current study established the involvement of MFG-E8 in antioxidant effects during neuroinflammation. It may achieve the effects through the regulation of Keap-1/Nrf-2/HO-1 pathways.

## 1. Introduction

Neuroinflammation plays an important role in the development of a set of neurological disorders, for example, cerebral infarction, Alzheimer's Disease (AD), and multiple sclerosis [1–4]. Microglia account for 5% of the total cells in the brain, and they are key cells in balancing neuroinflammation. Once activated, they would produce a set of factors, including tumor necrosis factor-*α* (TNF-*α*), interleukin-1*β* (IL-1*β*), interleukin-6 (IL-6), and reactive oxygen species (ROS) [5–7]. ROS accumulates from impaired degradation and/or excessive production in cells, in response to insults [8]. Microenvironment in the central nervous system turned imbalanced, and subsequent neuronal damage occurs [9]. Therefore, it is believed that effective modulation of microglial oxidative stress can be a therapeutic option for neurological disorders.

Milk fat globule-epidermal growth factor-factor 8 (MFG-E8) is a protein widely expressed throughout the body. It is initially identified in the mammary epithelia during lactation [10] and is attracting attention for its existence in the brain. MFG-E8 could modulate microglia activities through its connection with *α*_V_ *β*_3/5_ integrin [11]. Many studies have indicated its anti-inflammatory role in AD and ischemic stroke models [11, 12], reprograming microglia from a M1 (inflammatory) to a M2 phenotype (anti-inflammatory) [13]. However, the effects of MFG-E8 on microglial oxidant responses have not been established.

We investigate here the involvement of MFG-E8 in microglial oxidative stress. The findings would help establish a novel framework of a therapeutic option for neuroinflammation.

## 2. Materials and Methods

All the experiments and procedures were approved by the Animal Ethics Committee of Sun Yat-sen University and conducted in accordance with the Declaration of Helsinki (1964).

### 2.1. Chemical Agents and Antibodies

The lipopolysaccharide (LPS) and Glutathione (GSH) assay kits were purchased from Sigma-Aldrich Chemical Company (St. Louis, USA). DAPI was from Thermo Fisher Scientific. Mouse recombinant MFG-E8 protein was from R&D Systems (Minneapolis, USA). F12-Dulbecco's modified Eagle's medium (DMEM), fetal bovine serum (FBS), phosphate-buffered saline (PBS), and penicillin/streptomycin came from Life Technologies (New York, USA). The Iba-1 antibody was from Wako (Fuji, Japan). The TRIzol reagent was purchased from Invitrogen (Waltham, USA). Kelch-like ECH-associated protein 1 (Keap-1), Nuclear factor E2-related factor 2 (Nrf-2), Heme oxygenase 1 (HO-1), and GAPDH antibodies were purchased from Cell Signaling Technology (Beverly, USA). The Malondialdehyde (MDA) kit, Superoxide Dismutase (SOD) kit, ROS assay kit, TNF-*α* ELISA kit, and IL-6 ELISA kit were purchased from Beyotime (Shanghai, China).

### 2.2. Animal Treatment

Eight-week-old wild-type male C57 mice (25-30 g) were from the Animal Center of Sun Yat-sen University. Mice were kept in an environmental-controlled room (22°C ± 3°C, 12 h light-dark cycle, relative humidity of 60 ± 5%, and free access to food and drinking water were preserved). They were randomly divided into the following groups: (1) Control, daily intraperitoneal PBS injection for 7 days as a vehicle; (2) MFG-E8, daily intraperitoneal PBS (2 ml) and daily intracerebroventricular MFG-E8 (1 *μ*g) injection; (3) LPS, daily intraperitoneal PBS (2 ml)+LPS (250 *μ*g/kg) injection for 7 days; and (4) LPS+MFG-E8, daily intraperitoneal LPS (250 *μ*g/kg) and daily intracerebroventricular MFG-E8 (1 *μ*g) for 7 days. At the 8th day, the mouse brains were quickly collected after cardiac perfusion with PBS. The left or right hemispheres of the brain were randomly chosen for immunofluorescence staining and for the activity of ROS, MDA, SOD, and GSH, as well as for the detection in quantitative real-time PCR and western blot analysis.

### 2.3. Primary Microglial Culture and Treatment

Primary microglia were prepared using the brains of 1-3-day-old neonatal C57 mice, which were from the Animal Center of Sun Yat-sen University. Microglia were isolated and purified using a modified protocol reported in our previous studies [1, 13]. Microglia were treated with LPS (100 ng/ml) or MFG-E8 (100 ng/ml) for 1 h before sample collection. For the combined treatment, microglia were treated with MFG-E8 (100 ng/ml) for 1 h before LPS incubation.

### 2.4. Quantitative Real-Time PCR

Total RNA was extracted from brain tissues in each group using the TRIzol reagent, according to the standard instructions. Reverse transcriptase and quantitative PCR were performed on One-Step RT-PCR System (Applied Biosystems). The total RNA template (500 ng) was reverse-transcribed into cDNA using a PrimeScript RT Master Mix kit. Then, 2 ml of cDNA solution was turned into real-time polymerase chain reaction in a 20 *μ*l reaction volume with 10 *μ*l SYBR Premix Ex Taq II, 0.8 *μ*l PCR forward primer (0.4 mM), 0.8 *μ*l PCR reverse primer (0.4 mM), and 6 *μ*l ddH_2_O. Triplicate reactions were done for every sample. The primers of genes designed for the study are shown in Table 1.

### 2.5. MDA Assessment

For MDA determination, 100 *μ*l of cell suspension or tissue homogenate was mixed with 1 ml of solution containing thiobarbituric acid. The mixture was incubated at 95°C for 40 min and then cooled down using tap water. The samples were centrifuged at 4000 rpm for 10 min at room temperature. The absorbance of the supernatant was measured at 532 nm. The concentration of MDA was expressed as nanomoles of MDA per milligram of protein.

### 2.6. SOD Analysis

SOD activity was measured by using commercial kits according to the manufacturer's instructions (Nanjing JianCheng Bioengineering Institute, Nanjing, China). Briefly, for SOD activity determination, 20 *μ*l of cell suspension or tissue homogenate was mixed with 220 *μ*l of reaction solution, and the mixture was incubated at 37°C for 30 min. The absorbance was measured at 450 nm, and the results were recorded for analysis.

### 2.7. ROS Assay

The level of intracellular ROS was assessed using a ROS assay kit. The brain homogenate in each group was rendered as single cell suspension according to the protocol of Villalba et al. [14]. Then, cell suspension was added into a 96-well plate and incubated with 2′,7′-dichlorodihydrofluorescein diacetate (DCFH-DA) for 1 h. Primary cells were seeded in the 96-well plate and added with DCFH-DA for 1 h after treatment. Then, the fluorescent signal was assessed with an excitation at 488 nm and emission at 525 nm using a microplate reader. Fluorescent intensity was normalized to control wells for analysis.

### 2.8. GSH Detection

The GSH content was determined according to the manufacturer's protocol. Generally, GSH reacted with 5,5′-dithio-bis-2-nitrobenzoic acid to produce a yellow chromophore, and the absorbance was measured at 412 nm using a UV spectrometer.

### 2.9. Immunofluorescence Staining

For *in vivo* samples, they were fixed with 4% paraformaldehyde at 4°C for 24 h. The brains were dehydrated in a gradient sucrose solution (10%, 20%, and 30%), each at 4°C for 24 h. Brain sections were cut with a thickness of 10 *μ*m and then stored in cryoprotectant solution at 30°C until use. Sections were rinsed using PBS with 0.3% Triton X-100 for 3 times (each for 10 min) at room temperature and blocked in a solution with 5% normal goat serum for 1 h. After washing 3 times, sections were incubated with the Iba-1 antibody (1 : 500) for 24 h in a humidified chamber at 4°C. Then FITC-conjugated secondary antibody (1 : 200) was used to incubate samples for 1 h. Sections were coverslipped with glycerol and observed using a fluorescence microscope (Carl Zeiss, Germany).

For *in vitro* staining, microglia were seeded at 0.8 × 10^6^ on 1.5 mm^2^ coverslips for 24 h. After treatment, cells were fixed with ice-cold 4% paraformaldehyde for 20 min at 4°C. They were then air-dried followed by blocking and permeabilizing with 1% BSA and 0.1% Triton X-100 in PBS for 30 min. Then, cells were incubated with the Iba-1 antibody (1 : 500) for 24 h in a humidified chamber at 4°C. After washing three times using 0.3% Triton X-100 in PBS, the FITC-conjugated secondary antibody (1 : 200) was used to incubate samples for 1 h. Coverslips were transferred onto glass slides after 10 min staining with DAPI. Images were acquired by a fluorescence microscope (Carl Zeiss, Germany).

Images were quantitatively analyzed for microglial morphology using a grid-crossing method, modified based on the study by Luckoff et al. [15]. Briefly, cell morphology was analyzed by counting the number of grid-crossing points per cell in each group.

### 2.10. Western Blot Analysis

Microglia were scraped and lysed in a RIPA buffer at 4°C for 1 h. The total intracellular protein was extracted and quantified by a BCA kit according to the protocol from the suppliers. Cell lysates were solubilized with a SDS sample buffer (40 *μ*g/lane) and separated by 10% SDS-PAGE (110 V, 75 min). Proteins were then transferred to a 0.45 *μ*m PVDF membrane at 60 V for 1 h. After that, the membrane was blocked using TBST with 3% BSA. The following incubation with antibodies (anti-Keap-1, 1 : 1000; anti-Nrf-2, 1 : 1000; anti-HO-1, 1 : 1000; and anti-GAPDH, 1 : 500) was applied for 24 h at 4°C. Then, horseradish peroxidase- (HRP-) conjugated secondary antibodies were subsequently adopted for 1 h at room temperature and detected with the enhanced chemiluminescence (ECL) plus detection system. The density of each band was quantified by Quantity One Software. The density ratio represented the relative intensity of each band against GAPDH and normalized those in controls.

### 2.11. Statistical Analysis

Data in this study were depicted as the mean ± SD and analyzed using Statistical Package for the Social Sciences (SPSS) version 21.0. One-way analysis of variance (ANOVA) was used to analyze variables with the least significant difference (LSD). The level of statistical significance was set as *P* < 0.05.

## 3. Results

### 3.1. Effects of MFG-E8 on LPS-Induced Microglial Activation

**Figure 1:**
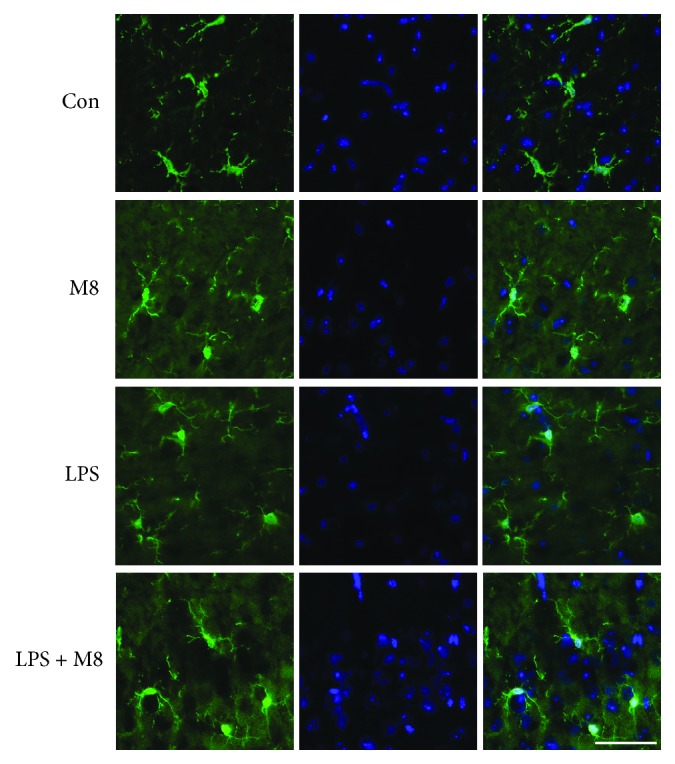
MFG-E8 inhibited LPS-induced microglial activation *in vivo*. Representative immunofluorescent staining images of Iba-1 (green) and DAPI (blue) in the cerebral cortex of mice of Control (*n* = 10), MFG-E8 (*n* = 10), LPS (*n* = 10), and LPS+MFG-E8 (*n* = 10). Bar = 50 *μ*m. Con: Control; M8: MFG-E8.

**Figure 2:**
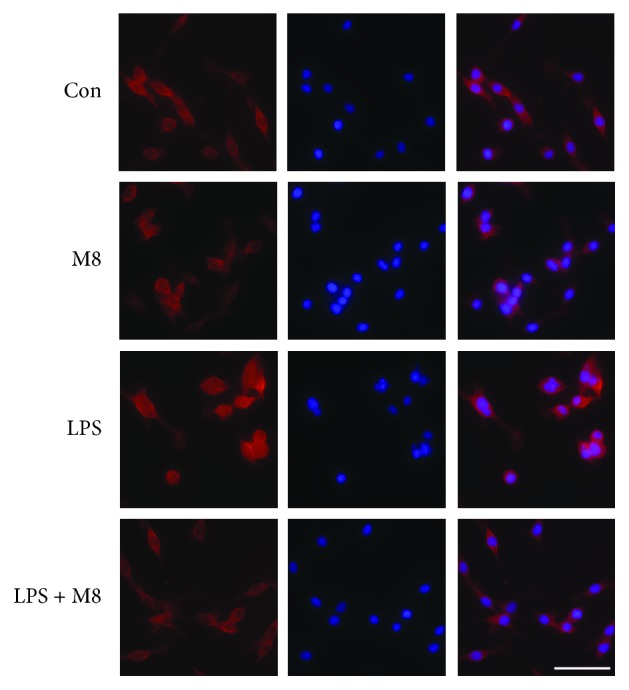
MFG-E8 inhibited LPS-induced microglial activation *in vitro* (*n* = 3). Representative immunofluorescent staining images of Iba-1 (red) and DAPI (blue) in primary microglia of Control, MFG-E8, LPS, and LPS+MFG-E8. Bar = 20 *μ*m. Con: Control; M8: MFG-E8.

**Figure 3:**
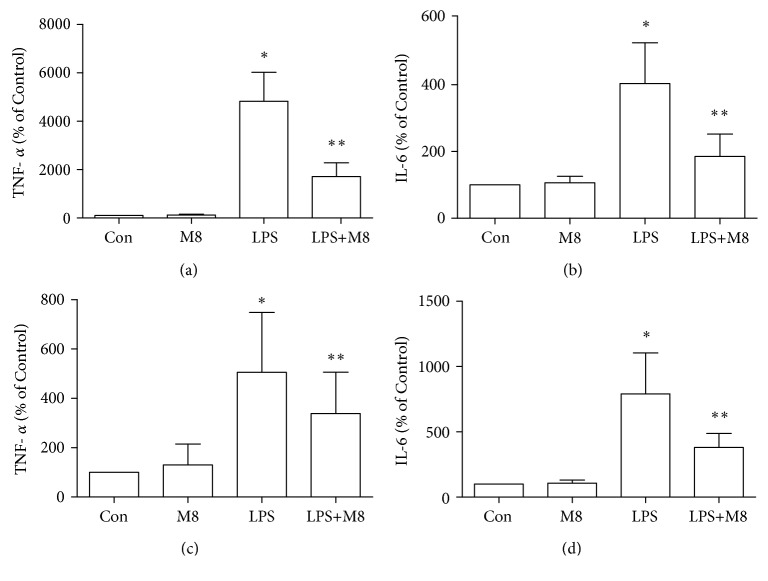
Effects of MFG-E8 on production of TNF-*α* and IL-6 in the cerebral cortex (a, b) and primary microglia (c, d) of Control, MFG-E8, LPS, and LPS+MFG-E8. Data were expressed as the mean ± SD (*in vivo*, *n* = 10; *in vitro*, *n* = 3). ^∗^ *P* < 0.05 versus the Control group; ^∗∗^ *P* < 0.05 versus the LPS group. Con: Control; M8: MFG-E8.

Microglial activation was observed through staining with an anti-Iba-1 antibody. As shown in *in vivo* results, microglia in controls showed a ramified form, while it turned, after LPS exposure, into an amoeba shape with a larger cell body and thicker processes, consistent with a deramified and amoeboid morphology (Figure 1). Brain sections from mice treated with MFG-E8 before LPS exhibited similar morphology with controls. We also found the consistent changes in each group *in vitro* (Figure 2). We further used the grid-crossing analysis method to quantify the morphology of cells (Supplementary Figure 1). It showed that cells in controls occupied more grids than cells treated with LPS, and the tendency by LPS was significantly reversed by MFG-E8 pretreatment. Production of inflammatory factors is a symbol of microglial activation. We assessed the levels of TNF-*α* and IL-6 after treatment (Figure 3). LPS induced higher levels of TNF-*α* and IL-6 in the brains (Figures 3(a) and 3(b)) and cellular culture (Figures 3(c) and 3(d)), which were inhibited by MFG-E8 preincubation. However, no obvious changes of *in vivo* and *in vitro* morphology and inflammatory factor production were seen when MFG-E8 was applied alone.

### 3.2. Effects of MFG-E8 on LPS-Induced Oxidative Stress *In Vivo*

**Figure 4:**
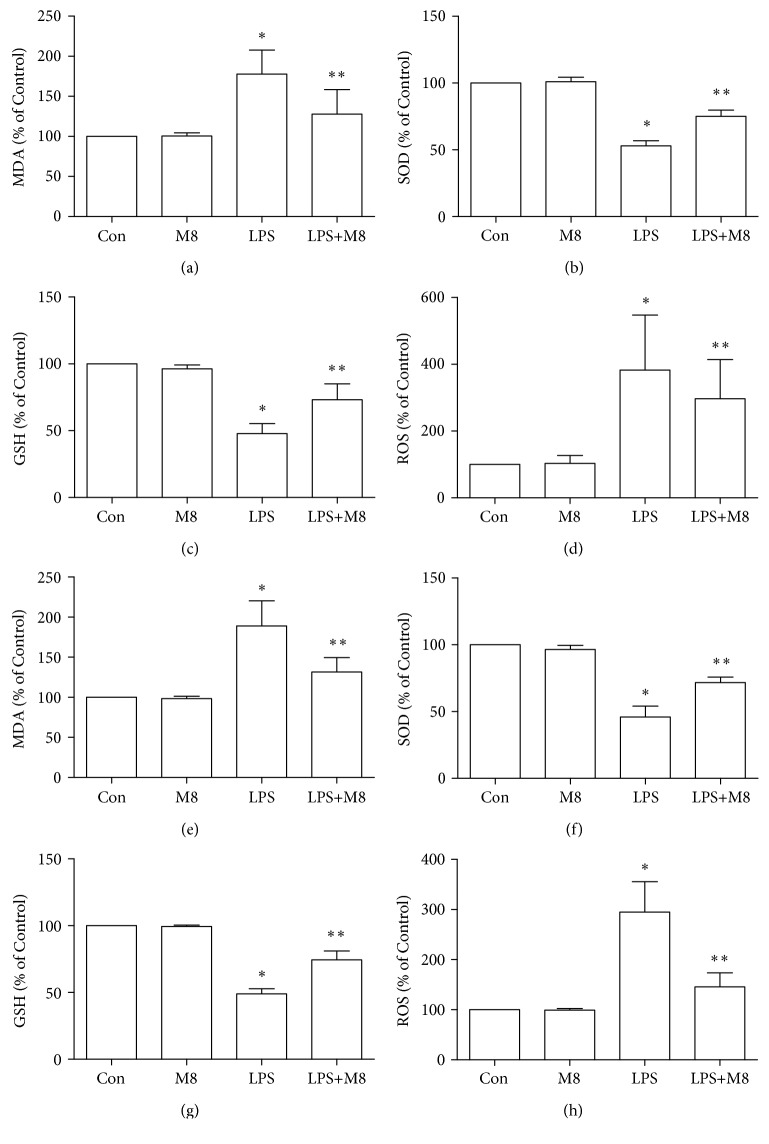
Effects of MFG-E8 on oxidative stress markers in the cerebral cortex (a–d) and primary microglia (e–h) of Control, MFG-E8, LPS, and LPS+MFG-E8. Data were expressed as percentage of Control. Data were expressed as the mean ± SD (*in vivo*, *n* = 10; *in vitro*, *n* = 3). ^∗^ *P* < 0.05 versus the Control group; ^∗∗^ *P* < 0.05 versus the LPS group. Con: Control; M8: MFG-E8.

Administration of LPS significantly increased MDA and ROS as compared to controls, whereas MFG-E8 reversed the increase of the MDA and ROS contents (Figures 4(a) and 4(d)). Antioxidative effects were evaluated by analyzing the parameters of SOD and GSH (Figures 4(b) and 4(c)). When LPS was applied, the SOD and GSH levels were suppressed significantly, which were blocked by MFG-E8. Also, MFG-E8 in the absence of LPS did not alter the trends of MDA, ROS, SOD, and GSH in the brain, compared with control mice.

### 3.3. Effects of MFG-E8 on Microglial Oxidative Stress in Response to LPS *In Vitro*

We assessed here oxidative stress in primary microglia. LPS induced a significant increase of MDA and ROS (Figures 4(e) and 4(h)) and decreased production of SOD (Figure 4(f)) and GSH (Figure 4(g)), compared with controls. However, MFG-E8 pretreatment tended to reverse the above changes against LPS. Moreover, the single use of MFG-E8 did not change the contents of MDA, ROS, SOD, and GSH in microglia, when compared with control cells.

### 3.4. Effects of MFG-E8 on Keap-1/Nrf-2/HO-1 Pathways

**Figure 5:**
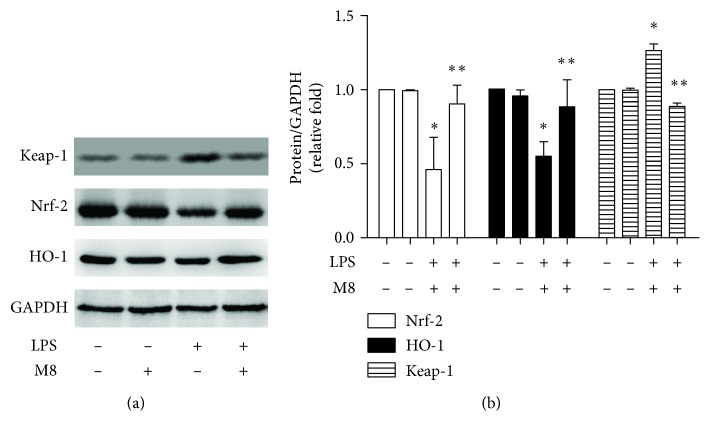
MFG-E8 reversed LPS-induced downregulation of Nrf-2/HO-1. (a) Representative western blot images of Nrf-2, HO-1, and GAPDH. (b) Quantitative analysis of each group was conducted. Fold changes of Nrf-2 (white bar) and HO-1 (black bar) expression were shown. Data were expressed as the mean ± SD (*n* = 3). ^∗^ *P* < 0.05 versus the Control group; ^∗∗^ *P* < 0.05 versus the LPS group. Con: Control; M8: MFG-E8.

**Figure 6:**
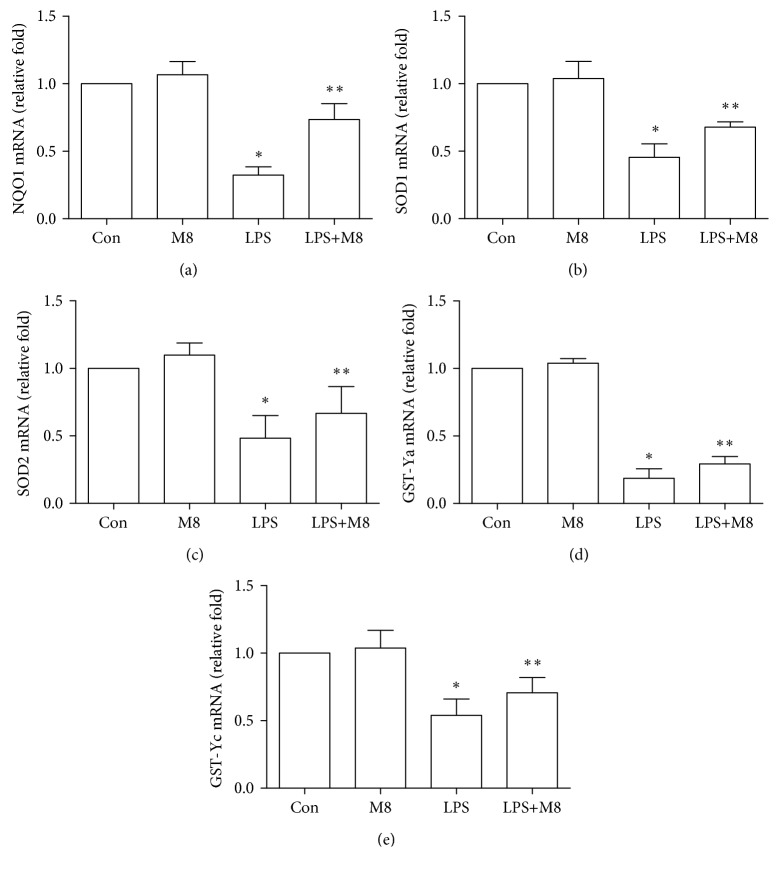
mRNA levels of NQO1, SOD1, SOD2, GST-Ya, and GST-Yc in primary microglia treated with LPS and MFG-E8. Data were expressed as the mean ± SD (*n* = 3). ^∗^ *P* < 0.05 versus the Control group; ^∗∗^ *P* < 0.05 versus the LPS group. Con: Control; M8: MFG-E8.

Keap-1/Nrf-2/HO-1 is an important pathway in cellular oxidative stress. We first assessed the expression of Keap-1, Nrf-2, and HO-1 in microglia. It indicated higher Keap-1 expression and lower levels of Nrf-2 and HO-1 in cells with LPS than controls (Figures 5(a) and 5(b)). However, MFG-E8 pretreatment significantly blocked the above changes by LPS. MFG-E8 treatment alone did not change the expression of these proteins. We also evaluated Nrf-2-regulated genes (Figure 6) and found that mRNAs of NQO1, SOD1, SOD2, GST-Ya, and GST-Yc were significantly suppressed by LPS, which were markedly reversed by MFG-E8.

## 4. Discussion

The current study evaluated the effects of MFG-E8 on microglial oxidative responses against LPS. We found oxidative stress in the mouse brain, and microglia were alleviated by MFG-E8 treatment, suggesting its involvement in neuroprotection. But MFG-E8 alone did not induce changes of oxidative responses. The specific mechanisms need further clarification.

Microglial cells are primary participants in neuroinflammation. Resting microglia exhibit surveillance function by sensing the extracellular changes with their branches [16]. In brain injury, microglia transformed into a specific morphology with motile processes and long branches, ensuring their activity in the sensation of external stimuli [17]. In this study, the cortex was chosen for both *in vivo* observation and *in vitro* microglial purification. We used the method previously used in 2017 [13] to provide primary microglia for *in vitro* treatment. This protocol ensures the high production of microglia and the inflammatory features in response to external factors. Studies indicated that cells from cortex regions present increased Iba-1 immunoreactivity after LPS challenge, which is usually used as a marker to detect microglial morphological changes [18, 19]. In the current study, LPS induced microglia into an activation morphology with an amoeba shape. MFG-E8 reversed the shape changes by LPS. To better demonstrate the immunostaining results, we used a grid-crossing method to quantify cellular morphology. This method calculated the grids covered per cell in each group and discovered more girds in MFG-E8-pretreated cells than in LPS-stimulated cells. We speculated that it was attributed to the role of MFG-E8 on a microglial cytoskeleton. It is believed that MFG-E8 is a cytoskeletal rearrangement enhancer for microglial phagocytosis [20, 21]. Neniskyte and Brown [11] demonstrated that MFG-E8 is an essential factor for microglial phagocytosis. Cells from MFG-E8-knockout animals exhibit deficits in phagocytic ability. Subsequent signaling pathways in microglia, which were associated with cytoskeletal rearrangement, for example, DOCK180/Rac-1 [22], get stimulated.

Microglial production of inflammatory factors is concomitant with the morphological changes during activation. In the previous study, we found that MFG-E8 pretreatment significantly reversed A*β*-induced microglial activation along with an upregulation of IL-1*β*, IL-6, and TNF-*α* [1, 13]. An anti-inflammatory phenotype (M2) was induced by MFG-E8 [13]. This is also confirmed by assessing TNF-*α* and IL-6 in LPS-treated cells in the study. Moreover, activated microglia show an imbalance in production and clearance of oxidative factors [23]. We first observed MDA and ROS overproduction in both the brain and microglial samples treated with LPS. Antioxidative factors, GSH and SOD, were decreased in the same samples. MFG-E8 pretreatment then suppressed the oxidative changes led by LPS, implying the role of MFG-E8 in oxidative balance. Nrf-2 is a pleiotropic protein and a key antioxidant sensor. The activation of this protein is crucial for cellar hemostasis [24–27]. Under physiological conditions, Nrf-2 is bound to its natural inhibitor Keap-1 in the cytoplasm. By insults and stimuli, Nrf-2 would be released by Keap-1, translocate into the nucleus, and mediate the transcription of HO-1, leading to resistance to oxidative stress [28]. The upregulation of Nrf-2 and HO-1, as well as the inhibition of Keap-1 by MFG-E8, indicated that MFG-E8 achieved its antioxidant effects via the Nrf-2/HO-1 pathway. Nrf-2-regulated genes, including NQO1, SOD1, SOD2, GST-Ya, and GST-Yc, were also increased by MFG-E8 against LPS, confirming the regulatory role of MFG-E8 on the Nrf-2 pathway. This broadened the understanding of MFG-E8, which is a promising therapeutic agent for neurological disorders.

Interestingly, MFG-E8 alone did not induce changes in the microglial state, while its antiactivation role was only seen in LPS-treated samples. Therefore, MFG-E8 might exhibit its protective effects in an inflammation environment, but not under normal conditions. Once injury occurs, the balanced environment changed along with an inflammatory change. In this case, MFG-E8 started to act its role on microglia. The underlying mechanisms should be explored in the future.

## 5. Conclusion

The current study tries to establish the antioxidant role of MFG-E8 on microglia. MFG-E8 is involved in antioxidant effects during neuroinflammation. It may achieve the effects through the regulation of Keap-1/Nrf-2/HO-1 pathways.

## Figures and Tables

**Table 1:** Primer sequences for real-time PCR.

	Forward 5′-3′	Reverse 5′-3′
NQO1	ATTGTACTGGCCCATTCAGA	GGCCATTGTTTACTTTGAGC
SOD1	ATCCACTTCGAGCAGAAG	TTCCACCTTTGCCCAAGT
SOD2	AGCGGTCGTGTAAACCTCA	AGACATGGCTGTCAGCTTC
GST-Ya	AAGCCAGGACTCTCACTA	AAGGCAGTCTTGGCTTCT
GST-Yc	GGAAGCCAGTCCTTCATTACT	CGTCATCAAAAGGCTTCCTCT
*β*-Actin	GTCAGAAGGACTCCTATGTG	CTCATTGTAGAAGGTGTGGT

## Data Availability

The data used to support the findings of this study are available from the corresponding author upon request.

## Supplementary Materials

Supplementary Materials Supplementary Figure 1: grid-crossing analysis of microglia morphology in animals and primary microglia.
