# Supplementary material for: Milk Fat Globule-Epidermal Growth Factor-Factor 8 Reverses Lipopolysaccharide-Induced Microglial Oxidative Stress
Source: Oxid Med Cell Longev. 2019 Mar 13;2019:2601394. doi: 10.1155/2019/2601394 (PMC6436360; doi:10.1155/2019/2601394)
Supplement: Supplementary Materials — Supplementary Figure 1: grid-crossing analysis of microglia morphology in animals and primary microglia. [file 2601394.f1.pdf]

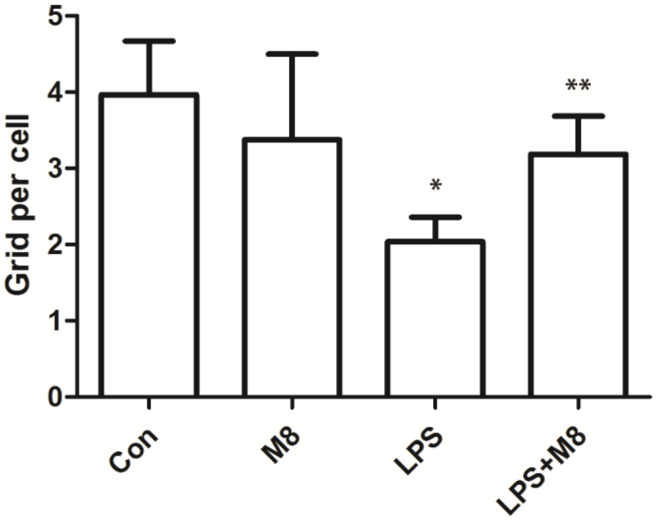


(a)

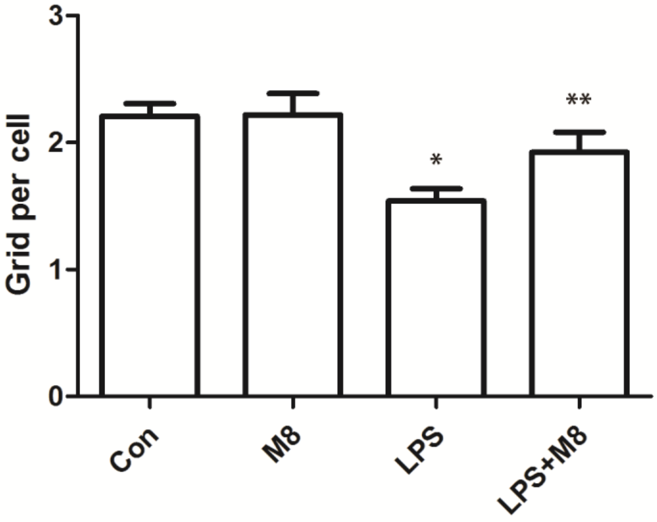


(b)

SUPPLEMENTAL FIGURE 1. Grid cross analysis of microglia morphology in animals (a) and primary microglia (b). Data were expressed as mean ± SD (*in vivo*, n = 10; *in vitro*, n = 3). * $P < 0.05$, versus Control group; ** $P < 0.05$, versus LPS group. Con, Control; M8, MFG-E8.
